# Effects of Different Selenium Sources on the Laying Performance, Egg Quality, Antioxidant, and Immune Responses of Laying Hens under Normal and Cyclic High Temperatures

**DOI:** 10.3390/ani12081006

**Published:** 2022-04-13

**Authors:** Weihan Wang, Ruifen Kang, Meiling Liu, Zhong Wang, Lihong Zhao, Jianyun Zhang, Shimeng Huang, Qiugang Ma

**Affiliations:** State Key Laboratory of Animal Nutrition, College of Animal Science and Technology, China Agricultural University, Beijing 100193, China; w18833192466@163.com (W.W.); ruifenkang@cau.edu.cn (R.K.); 17861509728@163.com (M.L.); wangzh@cau.edu.cn (Z.W.); lihongzhao100@126.com (L.Z.); jyzhang@cau.edu.cn (J.Z.); shimengh@cau.edu.cn (S.H.)

**Keywords:** trace element, laying hen performance, high temperature, inflammation, oxidative stress

## Abstract

**Simple Summary:**

Heat stress is a major environmental stressor in livestock production, which seriously threatens the global livestock industry and causes huge economic losses. Strategies to address heat stress are mainly divided into three categories: genetic regulation, environmental regulation, and nutritional diet regulation. Among them, nutritional regulation is one of the current research hotspots. Selenium is an essential trace element for animals and humans. As a component of some anti-oxidant enzymes and selenoproteins, it plays an important role in improving anti-oxidation, anti-stress, immune function, and reproduction performance. This study investigated the effects of different selenium sources on the laying performance, egg quality, antioxidant, and immune responses of laying hens under different temperatures. The results showed that supplementation with different selenium sources can increase the egg yolk color, antioxidant capacity, and immune status, and can alleviate heat stress damage. Additionally, the effect of organic selenium is better than that of inorganic selenium.

**Abstract:**

The aim of this study was to evaluate the effects of different selenium (Se) sources on the laying performance, egg quality, antioxidant, and immune responses of laying hens under different temperatures. In an 8-week experiment, a total of 480 44-week-old laying hens were randomly divided into 8 groups, with 6 replicates for each group and 10 hens per replicate, and fed with a basal diet (BK), basal diet with 0.3 mg/kg of Se from sodium selenite (SS), from Se yeast (SY), or from selenium-enriched yeast culture (SYC) under normal temperature (NT, 26 ± 2 °C) and cyclic high temperature (CHT, 26 ± 2 °C~33 ± 2 °C). CHT decreased the laying performance and serum levels of Se, immunoglobulin G (IgG), and interleukin-10 (IL-10), and significantly increased the serum free triiodothyronine (FT3), deiodinase-I (DI-I), and heat stress protein (HSPs) (*p* < 0.05). In addition, SYC increased the egg yolk color, and SS increased serum IgG level. SS, SY, and SYC reduced the level of interleukin-6 (IL-6) (*p* < 0.05). In conclusion, Se can increase egg yolk color, antioxidant capacity, and immune capacity under heat stress, and the effect of organic Se is better than that of inorganic Se.

## 1. Introduction

With the global climate crisis, the frequency of high temperature weather has increased, and heat stress has become one of the important factors restricting the production of laying hens [1,2]. The high temperature environment will cause changes in the physiological functions of laying hens such as White Leghorn hens and Hy-Line Brown, and reduce their egg production, egg weight, eggshell thickness, and reproductive performance, while also altering the immunity of laying hens [3,4,5]. Thus, it is more urgent to develop new strategies to diminish negative effects of heat stress caused by high temperature and lighten economic loss caused by heat stress.

Selenium (Se) is an essential trace element for animals and humans. As a component of some antioxidant enzymes and selenoproteins, it plays an important role in improving anti-oxidation, anti-stress, immune function, and reproduction performance [6]. In addition, Se deficiency could cause a variety of diseases in humans and animals, for example Kaschin–Beck in humans; exudative quality, muscular trophic disease, and pancreatic trophic atrophy in poultry [7,8]; and white muscle disease in sheep [9]. Therefore, it is important to supplement Se to foods and feeds to prevent Se-deficiency diseases.

Se yeast (SY) is an organic Se source with high Se content that is transformed by microorganisms [10]. As a feed supplement Se product, SY has significant advantages over inorganic Se sources [11]. It has been reported that the bioavailability and retention of organic Se is higher than that of inorganic Se in broiler chickens [12,13]. The addition of Se in feed will affect the Se content in eggs, and SY has a higher deposition efficiency for Se than inorganic Se [11]. Compared with inorganic Se, SY can significantly increase the daily feed intake and eggshell strength of laying hens [14]. SY can improve the oxidative stress caused by high temperature and enteric bacterial infection, and that may be due to the fact that SY improves the redox state of the chickens [15]. More importantly, SY has the characteristics of strong activity, low toxicity, high absorption rate, low environmental pollution, and wide safety range compared to inorganic Se [16,17]. Thus, the reasonable use of SY is essential to improve chicken production performance.

At present, most studies are about the effect of different levels of the same Se source in the diet on the production performance of laying hens [5,14,18]; there are few reports on whether adding Se to feed can resist the adverse consequences of laying hens caused by heat stress. The purpose of this study was to investigate the effects of different sources of Se in the diet on the laying performance, egg quality, anti-oxidation, immune function, and heat stress associated indicators of laying hens under normal temperature (NT) and cyclic high temperatures (CHT). We evaluated the feeding effect of different sources of Se under heat stress, and provide a theoretical basis for the utilization of Se in the production practice of laying hens.

## 2. Materials and Methods

### 2.1. Animal Experimental Ethics

The experiment was approved by the China Agricultural University Animal Care and Use Committee (A0041011202-1-1, Beijing, China).

### 2.2. Selenium Source

Common yeast culture and selenium-enriched yeast culture (SYC) were obtained from Hebei Feimote Biotechnology Co., Ltd., and both use the same yeast strain (preservation number: ACCC20060). The SYC was produced by solid fermentation of the yeast strains in a selenium-containing medium, and the Se content was 30 mg/kg; Se yeast (SY) named Alkosel (inactivated whole cell yeast containing 1000 mg/kg of organic Se, from Lallemand Inc., Montreal, QC, Canada). Sodium selenite premix (SS) containing 1% of inorganic Se was purchased from Hebei Yuanda Zhongzheng Biotechnology Co., Ltd. (Shijiazhuang, China). The Se content in the feed and serum were analyzed by using a fluorescence spectrophotometer (Hitachi 850, Hitachi Ltd., Tokyo, Japan) according to the protocol of Liao et al. [19].

### 2.3. Animals and Treatments

**Figure 1:**
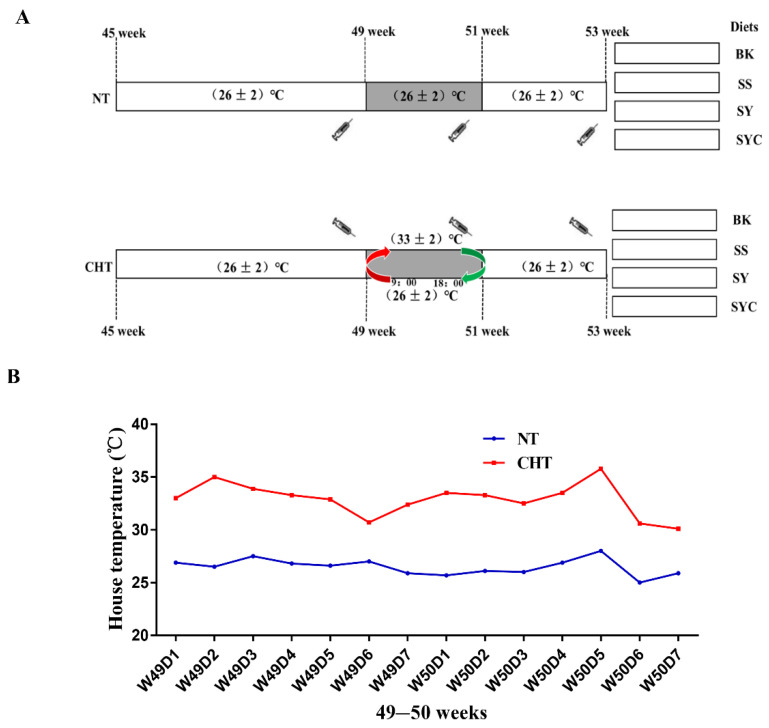
Experimental treatments and timeline of temperature control. (**A**) 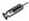: blood sampling; BK: basal diet; SS, SY, and SYC mean basal diet with 0.3 mg/kg of Se from sodium selenite, from Se yeast, and from selenium-enriched yeast culture, respectively; NT: normal temperature, the temperature maintained at (26 ± 2) °C from weeks 45 to 52, CHT: cyclic high temperature, the house temperature maintained at (26 ± 2) °C during the weeks of 45–48 and 51–52, however, from weeks 49 to 50, the house temperature was maintained at (33 ± 2) °C from 09:00 to 18:00, then cooled to (26 ± 2) °C. (**B**) Ambient temperature measured in the bird house from 9:00 to 18:00 during the period from 49 to 50 weeks. The letter W means week and D means day; for example, W49D1 means week 49 day 1.

A total of 480 44-week-old Peking Pink laying hens were purchased from Beijing Huadu Yukou Poultry Industry Co., Ltd. The chickens were housed in wire cages and randomly divided into 8 groups using a 4 × 2 double factorial design, with 6 replicates in each group of 10 birds each, with 2 hens per cage, which dimensions were 45 cm × 45 cm × 45 cm. Each cage was equipped with one nest and two nipples. The layer of normal temperature (NT) and cyclic high temperature (CHT) were reared in two houses with four groups of chickens in each house, and the temperatures were controlled by using an electric heating tube and a thermostat. The experimental hens were fed a basal diet without Se addition, or a basal diet with 0.3 mg/kg of Se from SS, from SY, or from SYC. The procedure of heat stress was as follows (Figure 1). During the 4-week normal feeding period (weeks 45–48), the house temperature was maintained at 26 ± 2 °C. Then, during the 2-week heat stress period (weeks 49–50), the house temperature was maintained at 33 ± 2 °C from 09:00 to 18:00, then cooled to 26 ± 2 °C, and maintained until 07:00 the next day, with ventilation for 2 min every 100 min. During the two-week convalescence (weeks 51–52), the house temperature was maintained at 26 ± 2 °C. NT groups keep the temperature at 26 ± 2 °C [4] during these three stages. A corn-soybean-meal diet was formulated as a basal diet according to Chicken Feeding Standards (except selenium) (NY/T33-2004). The diet composition and nutritional level are shown in Table 1. Feed and water were provided ad libitum during the 8-week experiment period. There was no mortality during the experiment.

### 2.4. Laying Performance

During the whole experimental period, the number of eggs and egg weight in each replicate were recorded daily. The feed consumption in each replicate was recorded weekly. The rate of egg production, mean weight of eggs, daily egg production, the ratio of feed:egg, and the average feed intake were then calculated.

### 2.5. Rectal Temperature

On the last day of normal feeding, heat stress, and convalescence (12:00 pm), two chickens were randomly selected from each repetition of each group to determine the rectal temperature by using a portable digital thermometer (Tianjin Jinming Instrument Co., Ltd., JM222L, Tianjin, China).

### 2.6. Egg Quality

On the last day of normal feeding, heat stress, and convalescence, three eggs were randomly selected from each repetition of each group to determine the egg quality. Egg quality mainly includes Haugh units (HU), egg yolk color, eggshell thickness, egg shell percent, and egg yolk percent. Egg HU and egg yolk color were determined by egg analyzer TM (Orka Teachnology Ltd., Ramat Hasharon, Israel). The eggshell thickness was calculated by a vernier caliper.

### 2.7. Blood Collection and Serum Analysis

One day before the end of normal feeding, heat stress, and convalescence, fasting for 12 h, two chickens were selected randomly for each replication, and wing vein blood sampling was performed. The blood samples were clotted in the heparin treated tubes (Jiangsu Rongsheng Jiamei Bioreagent Co., Ltd., Jiangsu, China) for 3 h and then centrifuged at 999× *g*, 20 min at 4 °C to obtain sera. The serum samples were stored at −20 °C until further analysis. Serum malondialdehyde (MDA, A003-1-2), glutathione peroxidase (GSH-Px, A005-1-2), vitamin E (VE, A008-1-1), nuclear factor-E2- related factor-2 (Nrf-2, KT81122-B), Kelch-like ECH-associated protein-1 (Keap-1, KT81121-B), immunoglobulin A (IgA, H108-1-2), immunoglobulin G (IgG, H106), immunoglobulin M (IgM, H109), interleukin-1β (IL-1β, H002), interleukin-6 (IL-6, H007-1-2), interleukin-10 (IL-10, H009-1), free triiodothyro- nine (FT3, H224), free thyroxine (FT4, H225), deiodinase-I (DI-I, KT7342-A), corticosterone (Cort, H205), and heat stress protein (HSPs, KT7345-A) were determined according to the manufacturer’s instructions of corresponding assay kits (Nanjing Jiancheng Bioengineering Institute, Nanjing, China). The samples were examined in duplicate and the results were measured using a microplatereader (MultiskanTM GO, ThermoFisher, Waltham, MA, USA).

### 2.8. Data Statistics and Analysis

The experimental data were analyzed using SPSS 20.0 software. The data before CHT were analyzed by a one-way ANOVA followed by Duncan’s multiple comparison, which was for normally distributed data to determine the differences in means among treatments, whereas the Kruskal-Wallis test was used for non-normally distributed data. After CHT, the data were analyzed according to a two-way ANOVA for a 2 × 4 factorial design using the general linear model (GLM) procedure. The difference of different Se sources and levels, temperatures, and their interactive effects were determined by Duncan’s multiple comparison. *p* < 0.05 was considered significant.

In the supplementary material, the relative fold change of indexes associated with rectal temperature, laying performance, egg quality, antioxidant, immune response, and heat stress were analyzed by using GraphPad Prism version 7.01 (GraphPad Software, Inc., San Diego, CA, USA). The relative fold change means the relative change fold of the period of heat stress (weeks 49–50) and convalescence (weeks 51–52) to normal feeding period (weeks 45–48). The data were analyzed by a one-way ANOVA followed by Duncan’s multiple comparison. Significance compared with weeks 45–48, * *p* < 0.05, ** *p* < 0.01, and *** *p* < 0.001; # indicated that weeks 51–52 were compared with weeks 49–50, # *p* < 0.05., ## *p* < 0.01, and ### *p* < 0.001.

## 3. Results

### 3.1. Rectal Temperature

As shown in Table 2, at week 48, compared with the BK, different Se sources had no significant effect on the rectal temperature (RT) of laying hens (*p* = 0.119), but Se supplementation significantly decreased the RT of laying hens (*p* < 0.05). At week 50, the RT increased significantly as the temperature increased (*p* < 0.05). In addition, the RT decreased in the SYC group compared to BK and SS (*p* < 0.05), but had no difference to SY (*p* > 0.05). At week 52, CHT significantly increased the RT of laying hens, compared with the normal temperature (NT) (*p* < 0.05); Se supplementation also had a significant effect on the RT of laying hens (*p* < 0.05). There were interactive effects between temperature and dietary Se sources and concentrations on RT at weeks 50 and 52 (*p* < 0.05). In addition, in NT, the RT of hens among BK, SS, SY, and SYC groups were decreased at week 50 compared to week 48, while the RT increased in week 52 compared to week 50 (*p* < 0.05). CHT significantly increased the RT among BK + HS, SS + HS, SY + HS, and SYC + HS groups during the heat stress period compared to the normal feeding period (*p* < 0.05), while the RT significantly decreased in the convalescence period compared to the heat stress period (*p* < 0.001) (Figure S1).

### 3.2. Laying Performance

As shown in Table 3, during the period from weeks 45 to 48, different Se sources and concentrations had no significant effect on the laying performance of laying hens (*p* > 0.05). From weeks 49 to 50, CHT significantly decreased the rate of egg production, daily egg production, mean weight of eggs, and the average feed intake, compared with the NT (*p* < 0.05). Different Se sources had no significant effect on the rate of egg production, daily egg production, mean weight of eggs, the average feed intake and the ratio of feed: egg of laying hens (*p* > 0.05). From weeks 51 to 52, CHT significantly decreased the rate of egg production, daily egg production, and mean weight of eggs and increased the ratio of feed:egg of laying hens compared with the NT (*p* < 0.05), but had no significant effect on the average feed intake (*p* > 0.05). Different Se sources had no significant effects, but had an upward trend in the rate of egg production, daily egg production, and average feed intake (*p* > 0.05). In addition, in NT, no obvious changes of laying performance were observed among BK, SS, SY, and SYC groups (*p* > 0.05). CHT significantly decreased the egg production and average feed intake among BK + HS, SS + HS, SY + HS, and SYC + HS groups during heat stress period compared to normal feeding (*p* < 0.001), while the egg production and average feed intake significantly increased in convalescence period compared with heat stress period (*p* < 0.01 and *p* < 0.001, respectively) (Figure S2).

### 3.3. Egg Quality

As shown in Table 4, during the period from 45 to 48 weeks, there was no obvious difference among different groups on the egg quality (*p* > 0.05). From weeks 49 to 50, CHT had no significant effects on the HU, the egg yolk color, eggshell thickness, eggshell percent, or egg yolk percent compared with the NT (*p* > 0.05). Se supplementation significantly increased the egg yolk color compared with the BK (*p* < 0.05). Different Se sources had a significant effect on eggshell percent and eggshell thickness (*p* < 0.05). From weeks 51 to 52, Se supplementation had a significant effect on egg yolk color; different Se sources had significant effects on egg yolk color and egg yolk percent (*p* < 0.05); the color of egg yolk in the SYC group was significantly darker than that of the SS and SY groups (*p* < 0.05). The egg yolk percent in SY was significantly higher than that in the SS group (*p* < 0.05). There were interactive effects between temperature and dietary Se sources on eggshell thickness from weeks 51 to 52 (*p* < 0.05). There were interactive effects between temperature and dietary Se concentrations on HU, egg yolk color, and eggshell thickness from weeks 51 to 52, and eggshell percent and egg yolk percent from weeks 45–48 (*p* < 0.05). In addition, in NT, the egg yolk color in the BK group decreased in both the weeks 49–50 and 51–52 compared to the weeks 45–48 (*p* < 0.001), while in SS and SYC groups, the color only decreased in week 51–52 compared to weeks 45–48 (*p* < 0.01 and *p* < 0.001, respectively), no obvious difference was observed in SYC group (*p* > 0.05). The eggshell thickness of week 51–52 significantly increased in BK and SS group compared to weeks 45–48 and 49–50 (*p* < 0.01), CHT significantly decreased egg yolk color among BK + HS, SS + HS, SY + HS, and SYC + HS groups during both the heat stress and convalescence period compared to normal feeding period (*p* < 0.05, *p* < 0.01, and *p* < 0.001, respectively) (Figure S3).

### 3.4. Serum Se and Vitamin E Content

As shown in Table 5, at weeks 48, 50, and 52, Se supplementation significantly increased the serum Se content compared with the BK (*p* < 0.05); no difference was observed among SS, SY, and SYC groups. In addition, CHT significantly decreased the serum Se content (*p* < 0.05) at weeks 50 and 52. The VE content in CHT was lower than that in NT at week 50 (*p* < 0.05), and SYC increased the VE content of layers at week 52 compared with the BK (*p* < 0.05). There were interactions between dietary Se levels and temperatures in Se content at week 52 and VE content at both weeks 48 and 50 (*p* < 0.05). Additionally, there were interactions between dietary Se sources and temperatures in VE content at weeks 48, 50, and 52 (*p* < 0.05). In addition, in NT, serum Se in BK group decreased in week 52 compared to week 48 and week 50 (*p* < 0.05 and *p* < 0.001, respectively). No obvious changes of Se were observed among SS, SY, and SYC groups (*p* > 0.05). Serum VE in BK and SYC group decreased in week 50 compared to week 48 (*p* < 0.01), and increased in BK in week 52 compared to week 50 (*p* < 0.001). Additionally, the VE level in SS group increased in week 52 compared to week 48 and week 50 (*p* < 0.001). No obvious changes of VE were observed in SY groups (*p* > 0.05). CHT significantly decreased the serum Se among BK + HS, SS + HS, SY + HS, and SYC + HS groups during heat stress period compared to normal feeding (*p* < 0.001), Furthermore, the serum Se among BK + HS, SS + HS, and SY + HS groups in week 52 were also lower than those in week 48 (*p* < 0.05 and *p* < 0.001, respectively), while no obvious changes of Se between week 48 and week 52 were observed the SYC + HS group (*p* > 0.05). CHT significantly decreased VE in the BK + HS group and increased VE in SYC + HS group in week 52 compared to week 48 (*p* < 0.05 and *p* < 0.001, respectively) (Figure S4).

### 3.5. Serum Antioxidant Status

As shown in Table 6, at week 48, compared with the BK, Se supplementation significantly decreased the serum MDA content and increased the serum GSH-PX level (*p* < 0.05), but had no significant effect on Nrf-2 and Keap-1 contents (*p* > 0.05). Compared to the BK, SS and SY significantly decreased MDA level (*p* < 0.05), and SS, SY, and SYC significantly increased GSH-PX level (*p* < 0.05). At week 50, compared with the BK, Se supplementation decreased serum MDA content (*p* < 0.05), and significantly increased GSH-PX level (*p* < 0.05), but had no significant effect on Nrf-2 (*p* > 0.05). At week 52, CHT increased the content of GSH-PX (*p* < 0.05), but had no significant effect on MDA, Nrf-2, or Keap-1, compared to the NT (*p* > 0.05). SS and SY significantly decreased serum MDA, and significantly increased GSH-PX level, compared with the BK (*p* < 0.05). In addition, in NT, the GSH-PX and Keap-1 in BK group decreased in both week 50 and 52 compared to week 48 (*p* < 0.05 and *p* < 0.01, respectively). Additionally, the GSH-PX in SYC group in week 52 was lower than that in week 48, and Keap-1 in SY and SYC decreased in both week 50 and 52 compared to week 48 (*p* < 0.05 and *p* < 0.001, respectively). No obvious difference of Keap-1 was observed in the SS group (*p* > 0.05). CHT significantly decreased GSH-PX level in the BK + HS group in both the heat stress period and convalescence period (*p* < 0.05 and *p* < 0.001, respectively); no obvious changes of GSH-PX were observed in the SS + HS, SY + HS, or SYC + HS groups (*p* > 0.05). Additionally, CHT significantly decreased Keap-1 in the BK + HS and SY + HS groups in the convalescence period compared to the normal feeding period (*p* < 0.01 and *p* < 0.001, respectively), and increased the MDA level among BK + HS, SS + HS, and SY + HS groups compared to normal feeding. No obvious changes of GSH-PX in the SS + HS and SY + HS groups of MDA in SYC + HS group were observed (*p* > 0.05) (Figure S5).

### 3.6. Serum Immune Indexes

As shown in Table 7, at week 48, SYC significantly decreased serum IL-6 level compared with the BK and SY (*p* < 0.05), but had no significant difference with SS (*p* > 0.05), while the level of IgM in SYC was significantly higher than that in SS (*p* < 0.05). At week 50, CHT significantly decreased serum IgG compared with the BK (*p* < 0.05), but had no significant effect on IgA, IgM, IL-1β, IL-6, and IL-10 (*p* > 0.05). SY significantly increased the level of IgM compared to SYC (*p* < 0.05). At week 52, CHT significantly decreased IL-1β and IL-10 in the sera compared with the NT (*p* < 0.05), but had no significant effect on IgA, IgG, IgM, or IL-6 (*p* > 0.05). Se supplementation significantly decreased IL-6 content (*p* < 0.05), and SS significantly increased serum IgG compared with the BK (*p* < 0.05). In addition, in NT, the IL-10 in the BK group decreased in week 50 compared to week 48 (*p* < 0.001), and increased in week 52 compared to week 50 (*p* < 0.001). No obvious changes of IgA, IgG, IgM, IL-1β, or IL-6 were observed in the BK group (*p* > 0.05). The levels of IgM in SYC group in week 52 increased compared to week 50 (*p* < 0.001), and the level of IL-6 decreased in SY group in both week 50 and 52 compared to week 48 (*p* < 0.05). CHT reduced the levels of IgG in BK + HS and SYC + HS in the heat stress period compared to normal feeding period (*p* < 0.05) and also decreased the level of the IL-10 in BK + HS, SS + HS, and SYC + HS groups (*p* < 0.01 and *p* < 0.001, respectively). No obvious changes of IgG in the SS + HS and SY + HS groups, or of IL-10 in the SY + HS groups, were observed (*p* > 0.05) (Figure S6).

### 3.7. Serum Heat Stress Associated Indexes 

As shown in Table 8, at week 48, different Se sources had no significant effects on the serum heat stress associated indexes (*p* > 0.05). At week 50, compared with the BK, different Se sources had no significant effects on HSPs, FT3, FT4, DI-I, and Cort (*p* > 0.05). CHT significantly increased the serum FT3 content of laying hens compared with the NT (*p* < 0.05), but had no significant effect on HSPs, FT4, DI-I, and Cort (*p* > 0.05). At week 52, CHT significantly increased the levels of serum DI-I and HSPs (*p* < 0.05), but had no significant effect on FT3, FT4, and CORT compared to NT (*p* > 0.05). The levels of DI-I in SY and SYC groups were significantly higher than that in SS (*p* < 0.05). In addition, SY significantly increased the content of FT3 compared with the BK and SYC (*p* < 0.05). There were interactions between dietary Se sources and temperatures in DI-I and FT4 at week 52 (*p* < 0.05); additionally, there were interactions between dietary Se sources and temperatures in CORT at week 50 (*p* < 0.05). In addition, in NT, the CORT in BK group decreased in both week 50 and 52 compared to week 48 (*p* < 0.05 and *p* < 0.01, respectively). The HSPs in BK in week 50 increased compared to week 48 (*p* < 0.01) and decreased in week 52 compared to week 50 (*p* < 0.001). No obvious changes of FT3, FT4, and DI-I were observed in BK group (*p* > 0.05). In the heat stress period, CHT increased the levels of FT3 in SY + HS and SYC + HS groups (*p* < 0.05 and *p* < 0.001, respectively), FT4 in SS + HS and SY + HS groups (*p* < 0.05 and *p* < 0. 01, respectively), and HSPs among BK + HS, SS + HS, SY + HS, and SYC + HS groups compared to normal feeding period (*p* < 0.001). Compared to the heat stress period, the levels of FT3 and FT4 were significantly decreased in SYC + HS group (*p* < 0.05) and SS + HS group (*p* < 0.01), respectively (Figure S7).

## 4. Discussion

### 4.1. Effects of Different Se Sources on Laying Performance of Heat-Stressed Laying Hens

High temperature is the main environmental factor affecting poultry production. Adult layer hens are prone to stress response due to plump surface feathers and underdeveloped sweat glands. Four weeks of continuous heat stress (35°C, 7 h/d) in 24-week-old Hy-Line Brown hens significantly decreased shell weight, feed intake, egg production, and body weight [20]. A previous study reported that heat stress reduced the feed conversion rate of laying hens by 31.6%, egg production by 36.4%, and egg weight by 3.41% [21]. The addition of organic selenium to the diet had no effect on the total weight, the white weight, shell weight, and shell thickness of eggs, but significantly increased egg yolk’s weight [22]. In the present study, the rate of egg production, daily egg production, and mean weight of eggs of laying hens were significantly decreased after challenging with CHT, which was similar to previous studies [23,24], but the ratio of feed: egg was significantly increased during week 51–52. This may be due to the increase in endogenous loss in layer hens repairing damage caused by CHT in weeks 51–52. A previous study has found that high temperature can increase the consumption of endogenous nutrients in pigs to compensate for the intestinal damage caused by high temperature [25]. Therefore, we speculated that the increase in feed-to-egg ratio during the recovery period after heat stress may also be caused by the increased consumption of endogenous nutrients during the heat stress period for alleviating heat stress injury. In addition, adding inorganic or organic selenium had no significant effect on egg weight, egg shell thickness, or egg yolk percentage, which was partially inconsistent with the study of Januzi. This may be due to the different types and ages of laying hens.

### 4.2. Effects of Different Se Sources on Egg Quality of Heat-Stressed Laying Hens

Eggs were considered as a complete food and an excellent source of fatty acids, vitamins, minerals, and essential amino acids. High temperature (33.3 °C) can significantly reduce the protein height and shell weight of eggs, as well as the weight of egg yolks [26]. When laying hens were exposed to heat stress, egg weight, egg shell weight, egg shell thickness, and specific gravity will be significantly decreased [1]. Reduced feed intake was considered the main factor leading to lower productivity of birds under high temperatures [27], which was in line with the present study. The addition of SY and SS to layer diets had no significant effect on the HU of eggs. SY significantly increased egg yolk color [28]. In the present study, compared with the selenium-free diet group, SYC significantly increased the yolk color, which was consistent with the study of Paton et al.

### 4.3. Effects of Different Se Sources on Serum Antioxidant Status of Heat-Stressed Laying Hens

Animals in a high temperature environment will produce excessive free radicals, including reactive oxygen radicals (ROS), which disrupt the body’s antioxidant system and cause oxidative stress in animals [29,30]. MDA can be used to evaluate the degree of lipid oxidative damage. GSH is an important antioxidant in the antioxidant system. It can not only remove peroxides in the enzymatic antioxidant system, but also help maintain the antioxidant capacity of vitamin C and vitamin E in the non-enzymatic antioxidant system [31]. The addition of inorganic Se and bacterial organic Se to the diet significantly increased serum Se levels and GSH-Px activity, and decreased MDA content of broilers [32]. Adding Se to the diet increased serum GSH-Px activity and liver SOD activity [33]. In the present study, the levels of GSH-PX and Se decreased with age. CHT also decreased the levels of GSH-PX and Se, and increased the level of MDA; however, Se supplementation could alleviate these adverse effects, which may be due to the Se being involved in the composition of glutathione peroxidase [34]. The CHT activated the body’s antioxidant system, further promoting the synthesis of glutathione peroxidase and decreasing the level of serum Se. In addition, compared with selenium-free diets, SS, SY, and SYC significantly increased serum GSH-Px, VE, and Se level, and significantly decreased MDA content, which was consistent with the previous study. It showed that the body activated its own antioxidant defense system to resist the harm caused by CHT to the body, and Se improved the body’s antioxidant capacity and can alleviate the body’s oxidative damage.

### 4.4. Effects of Different Se Sources on Immune Function of Heat-Stressed Laying Hens

It has been reported that increased ambient temperature will reduce the performance and immune status of poultry [35]. A study found that the ratio of circulating antibodies IgG and IgM in chickens decreased when exposed to heat stress [36]. The change of serum immunoglobulin content is an important indicator of animal immune function. It has been reported that heat stress can reduce the level of serum immunoglobulin, thymus, and spleen weight of broiler chickens [37]. The supplementation of 0.3 mg of Se to the diet significantly increased the total IgY titer and gammaglobulins in the sera of broilers, as well as improving humoral immunity [38]. A study found that Se-enriched earthworm powder with 1.0 mg/kg of Se increased the serum albumin and IgG of laying hens [5]. In the present study, compared to the NT group, CHT significantly decreased the serum IgG content; adding SS, SY, and SYC increased the immunity of layers, which was consistent with previous studies. It showed that Se improved the body’s immune function and can alleviate the body’s damage caused by heat stress.

### 4.5. Effects of Different Se Sources on Serum Heat Stress Associated Indexes of Heat-Stressed Laying Hens

The central nervous system regulates the secretion of hormones and responds to external stimuli. Cort is a key indicator of the strength of stress. Previous studies have reported that, during heat stress, it stimulates the hypothalamus to synthesize corticotropin-releasing hormone (CRH), the release of corticotropin (ACTH) from the anterior pituitary, and the adrenal cortex cells to secrete Cort to achieve the purpose of alleviating stress [39,40,41]. When chickens were under heat stress, the hypothalamic-pituitary-adrenal (HPA) axis was activated, and the content of Cort in the sera increased [42]. In the present study, compared to the NT group, CHT increased the Cort content, though not significantly; the trend was consistent with previous studies. In addition, the temperature of an animal’s body is also regulated by thyroid hormone, triiodothyroxine (T3), and thyroxine (T4). We found that the FT3 concentration significantly increased at week 50, which was in partial agreement with the report, which reported that after 14 days of continuous heat stress (35 °C) in 4-week-old broiler, the levels of FT3 and FT4 significantly increased [43], while the FT4 level had no significance in the present study. This difference may be related to the species, ages, and environment of the animals. When exposed to high temperatures, the body protects itself by synthesizing HSPs. The high concentrations of HSP70 can be found when poultry is exposed to heat stress. In the present study, compared to NT, CHT significantly increased the HSPs at week 52, which was consistent with the previous study. In addition, adding SS, SY, and SYC decreased the concentration of HSPs, which means that Se plays a certain role in helping the body recover from heat stress.

## 5. Conclusions

In conclusion, this study demonstrated that circulating high temperature (26 ± 2 °C~33 ± 2 °C) had no significant effect on the egg quality of laying hens, but significantly decreased the laying performance, serum IgG, and antioxidant capacity. Adding different Se had no obvious effect on laying performance, but can remarkably increase the color of egg yolk. In addition, Se supplementation significantly increased antioxidant capacity and serum IgG, further alleviating damage caused by heat stress.

## Figures and Tables

**Table 1:** Diet composition and nutritional levels.

	BK	SS	SY	SYC
Ingredients	Composition (%)			
Corn	63.93	63.93	63.93	63.93
Soybean meal	23.60	23.60	23.60	23.60
Ordinary yeast culture	1.00	1.00	1.00	0.00
Selenium-enriched yeast culture	0.00	0.00	0.00	1.00
Limestone	9.00	9.00	9.00	9.00
Dicalcium Phosphate	1.60	1.60	1.60	1.60
Salt	0.30	0.30	0.30	0.30
DL-methionine	0.11	0.11	0.11	0.11
L-lysine hydrochloride	0.08	0.08	0.08	0.08
Threonine	0.02	0.02	0.02	0.02
Tryptophan	0.02	0.02	0.02	0.02
Vitamins ^1^	0.04	0.04	0.04	0.04
Trace minerals ^2^	0.30	0.30	0.30	0.30
Nutrient	Levels			
ME ^3^, MJ/kg	14.91	14.91	14.91	14.91
Crude protein, %	15.40	15.40	15.40	15.40
Calcium, %	3.64	3.64	3.64	3.64
Phosphorus, %	0.35	0.35	0.35	0.35
Lysine, %	0.81	0.81	0.81	0.81
Total sulfur amino acids %	0.58	0.58	0.58	0.58
Threonine, %	0.61	0.61	0.61	0.61
Selenium ^4^, mg/kg	0.049	0.352	0.373	0.368

BK: basal diet, SS: sodium selenite, SY: selenium yeast, and SYC: selenium-enriched yeast culture. ^1^ The vitamins provided per kg of diet: vitamin A, 8000 IU; vitamin D3, 3600 IU; vitamin E, 21 IU; vitamin K3, 4.2 mg; vitamin B1, 3 mg; vitamin B2, 10.2 mg; folic acid, 0.9 mg; calcium pantothenate, 15 mg; smoke acid, 45 mg; vitamin B6, 5.4 mg; vitamin B12, 0.024 mg; and biotin, 0.15 mg. ^2^ The trace minerals provided per kg of diet: iron, 60 mg; manganese, 60 mg; copper, 8 mg; zinc, 80 mg; and iodine, 0.35 mg. ^3^ ME: metabolizable energy. ^4^ Except for the selenium content, all others are calculated values.

**Table 2:** Effects of different Se sources in diet on rectal temperature of laying hens.

Diets	HS ^1^	Rectal Temperature (°C)		
		Week 48 ^2^	Week 50 ^2^	Week 52 ^2^
BK	−	40.13	39.92	40.02
BK	+	40.15	40.80	40.18
SS	−	40.08	39.90	40.05
SS	+	40.08	40.77	40.12
SY	−	40.15	39.85	40.00
SY	+	40.07	40.73	40.12
SYC	−	40.13	39.78	40.02
SYC	+	40.07	40.68	40.10
SEM		0.026	0.035	0.036
BK		40.14	40.36 ^a^	40.10
SS		40.11	40.33 ^a^	40.08
SY		40.11	40.30 ^ab^	40.06
SYC		40.08	40.23 ^b^	40. 06
SEM		0.018	0.024	0.026
	–	40.11	39.86 ^b^	40.02 ^b^
	+	40.10	40.75 ^a^	40.13 ^a^
SEM		0.013	0.017	0.018
*p*-values	HS	0.651	<0.001	<0.001
	Se L	0.048	0.640	0.015
	Se S	0.364	0.872	0.771
	Se L × HS	0.207	<0.001	<0.001
	Se S × HS	0.453	<0.001	0.004

Different letters indicate statistically significant differences (*p* < 0.05). BK: basal diet, SS: sodium selenite, SY: selenium yeast, SYC: selenium-enriched yeast culture, Se L: selenium levels, and Se S: selenium sources. ^1^ HS− = Normal temperature (26 ± 2 °C), HS+ = Circulating high temperature (26 ± 2 °C~33 ± 2 °C). ^2^ Weeks 48, 50, and 52: each group has six replicates (*n* = 6).

**Table 3:** Effects of different Se sources in diets on performance of laying hens.

Diets	HS ^1^	Egg Production Rate (%)			Egg Production (g/hen/d)			Mean Weight of Eggs (g)			Average Feed Intake (g)			Feed: Egg Ratio (g:g)		
		Weeks 45–48 ^2^	Weeks 49–50 ^2^	Weeks 51–52 ^2^	Weeks 45–48	Weeks 49–50	Weeks 51–52	Weeks 45–48	Weeks 49–50	Weeks 51–52	Weeks 45–48	Weeks 49–50	Weeks 51–52	Weeks 45–48	Weeks 49–50	Weeks 51–52
BK	−	87.38	82.73	87.44	50.54	48.76	51.32	57.83	58.90	58.69	106.00	106.59	108.02	2.09	2.19	2.11
BK	+	91.91	71.66	82.03	53.52	40.21	46.59	58.24	56.12	56.81	106.38	83.11	103.94	1.99	2.09	2.24
SS	−	88.09	83.92	84.70	51.27	49.31	49.66	58.18	58.77	58.66	103.85	106.45	104.03	2.03	2.16	2.10
SS	+	90.00	72.61	84.12	52.56	40.94	48.85	58.39	56.43	58.06	111.04	87.33	106.56	2.12	2.14	2.19
SY	−	88.81	87.99	90.73	51.09	52.83	53.03	57.53	59.97	58.42	107.38	109.87	109.67	2.10	2.10	2.07
SY	+	88.81	73.46	85.64	51.43	41.87	48.55	57.92	56.99	56.67	111.09	81.67	107.81	2.16	1.97	2.24
SYC	−	89.76	87.26	89.63	51.33	50.77	51.83	57.20	58.20	57.83	106.14	107.07	105.62	2.06	2.11	2.04
SYC	+	90.00	75.59	85.55	51.59	42.00	48.68	57.32	55.53	56.88	107.81	83.41	108.54	2.09	2.04	2.25
SEM		1.420	2.222	1.918	0.898	1.481	1.131	0.359	0.790	0.444	2.044	2.01	2.109	0.047	0.066	0.048
BK		89.64	77.20	84.74	52.03	44.49	48.96	58.04 ^a,b^	57.52	57.75	106.19	94.86	105.98	2.04	2.14	2.18
SS		89.05	78.27	84.41	51.92	45.13	49.26	58.29 ^a^	57.61	58.36	107.45	96.89	105.29	2.07	2.15	2.15
SY		88.81	80.73	88.19	51.26	47.36	50.79	57.73 ^a,b^	58.48	57.55	109.24	95.78	108.74	2.13	2.03	2.16
SYC		89.88	81.43	87.59	51.46	46.39	50.26	57.26 ^b^	56.87	57.36	106.98	95.24	107.08	2.08	2.08	2.15
SEM		0.500	1.571	1.356	0.320	1.047	0.800	0.132	0.559	0.314	0.750	1.421	1.491	0.017	0.047	0.034
	−	88.51	85.48 ^a^	88.13 ^a^	51.06	50.42 ^a^	51.46 ^a^	57.69	58.96 ^a^	58.41 ^a^	105.85	107.49 ^a^	106.83	2.08	2.15 ^a^	2.08 ^b^
	+	90.18	73.34 ^b^	84.34 ^b^	52.28	41.26 ^b^	48.17 ^b^	57.97	56.27 ^b^	57.11 ^b^	109.08	83.88 ^b^	106.71	2.09	2.04 ^b^	2.23 ^a^
SEM		0.078	1.111	0.959	0.449	0.741	0.566	0.179	0.395	0.222	1.022	1.005	1.054	0.024	0.023	0.024
*p*-values	HS	0.104	<0.001	0.008	0.062	<0.001	<0.001	0.271	<0.001	<0.001	0.051	<0.001	0.936	0.691	0.049	<0.001
	Se L	0.733	0.113	0.209	0.516	0.144	0.224	0.352	0.833	0.982	0.315	0.502	0.542	0.187	0.348	0.539
	Se S	0.731	0.339	0.120	0.754	0.331	0.395	0.024	0.137	0.069	0.512	0.705	0.273	0.408	0.220	0.952
	Se L × HS	0.107	0.696	0.494	0.118	0.737	0.305	0.775	0.926	0.283	0.261	0.955	0.134	0.039	0.787	0.759
	Se S × HS	0.766	0.732	0.473	0.817	0.646	0.271	0.932	0.922	0.425	0.402	0.091	0.460	0.817	0.720	0.458

Different letters indicate statistically significant differences (*p* < 0.05). BK: basal diet, SS: sodium selenite, SY: selenium yeast, SYC: selenium-enriched yeast culture, Se L: selenium levels, and Se S: selenium sources. ^1^ HS− = Normal temperature (26 ± 2 °C), HS+ = Circulating high temperature (26 ± 2 °C~33 ± 2 °C). ^2^ Weeks 45–48, 49–50, and 51–52: each group has six replicates (*n* = 6).

**Table 4:** Effects of different Se sources in diets on egg quality of laying hens.

Diets	HS ^1^	Egg Haugh Unites			Egg Yolk Color			Eggshell Percent (%)			Egg yolk Percent (%)			Eggshell Thickness (mm)		
		Weeks 45–48 ^2^	Weeks 49–50 ^2^	Weeks 51–52 ^2^	Weeks 45–48	Weeks 49–50	Weeks 51–52	Weeks 45–48	Weeks 49–50	Weeks 51–52	Weeks 45–48	Weeks 49–50	Weeks 51–52	Weeks 45–48	Weeks 49–50	Weeks 51–52
BK	−	67.71	73.08	64.50	4.55	3.33	3.38	10.43	10.25	10.75	27.54	27.31	27.05	0.39	0.39	0.44 ^a^
BK	+	70.51	73.86	72.49	4.66	3.33	2.89	10.05	10.53	10.90	28.70	28.28	28.34	0.39	0.38	0.41 ^a,b^
SS	−	68.17	68.41	74.56	4.50	3.88	2.77	10.28	10.15	10.89	27.63	27.90	26.75	0.38	0.38	0.41 ^a,b^
SS	+	72.29	74.89	67.38	4.67	3.89	3.66	10.16	10.03	10.77	26.49	26.62	26.79	0.39	0.37	0.43 ^a^
SY	−	66.24	73.96	68.23	4.56	4.44	3.66	10.24	10.46	10.60	28.18	28.39	28.03	0.38	0.39	0.40 ^a,b^
SY	+	72.28	74.75	68.97	4.83	3.72	3.88	10.60	10.65	10.96	27.59	27.41	28.78	0.40	0.39	0.43 ^a^
SYC	−	67.08	73.73	69.73	4.44	4.00	3.89	10.25	10.47	10.78	28.24	27.52	28.26	0.38	0.38	0.39 ^b^
SYC	+	71.14	77.35	66.87	4.56	3.94	3.83	10.54	10.42	11.15	27.48	28.34	27.03	0.40	0.41	0.43 ^a^
SEM		2.299	2.310	2.987	0.130	0.223	0.245	0.166	0.169	0.191	0.405	0.459	0.526	0.006	0.007	0.008
BK		69.11	73.47	68.50	4.61	3.33 ^b^	3.14 ^b^	10.24	10.39	10.82	28.12 ^a^	27.80	27.69 ^a,b^	0.39	0.38	0.42
SS		70.23	71.66	70.98	4.58	3.88 ^a^	3.22 ^a,b^	10.22	10.09	10.83	27.07 ^b^	27.26	26.77 ^b^	0.39	0.37	0.42
SY		69.26	74.36	68.60	4.70	4.08 ^a^	3.78 ^a,b^	10.42	10.56	10.78	27.89 ^a,b^	27.90	28.41 ^a^	0.39	0.39	0.42
SYC		69.11	75.55	68.30	4.50	3.97 ^a^	3.86 ^a^	10.39	10.44	10.96	27.87 ^a,b^	27.93	27.64 ^a,b^	0.39	0.39	0.41
SEM		0.819	1.633	2.112	0.045	0.158	0.173	0.059	0.119	0.135	0.174	0.325	0.372	0.004	0.005	0.005
	−	67.29 ^b^	72.30	69.26	4.51	3.92	3.43	10.30	10.33	10.75	27.90	27.78	27.52	0.38	0.38	0.41
	+	71.55 ^a^	75.22	68.93	4.68	3.72	3.57	10.33	10.41	10.94	27.57	27.67	27.74	0.39	0.39	0.42
SEM		1.150	1.155	1.494	0.065	0.112	0.122	0.083	0.084	0.095	0.228	0.230	0.263	0.003	0.004	0.004
*p*-values	HS	0.012	0.082	0.879	0.077	0.226	0.428	0.759	0.529	0.166	0.304	0.771	0.566	0.006	0.342	0.077
	Se L	0.823	0.840	0.746	0.862	<0.001	0.021	0.453	0.870	0.837	0.173	0.792	0.836	0.815	0.858	0.207
	Se S	0.869	0.237	0.622	0.333	0.683	0.025	0.422	0.023	0.615	0.135	0.265	0.013	0.627	0.023	0.152
	Se L × HS	0.609	0.454	0.028	0.722	0.485	0.040	0.044	0.325	0.848	0.011	0.059	0.103	0.147	0.506	<0.001
	Se S × HS	0.887	0.475	0.422	0.816	0.210	0.153	0.309	0.623	0.346	0.825	0.056	0.177	0.907	0.024	0.481

Different letters indicate statistically significant differences (*p* < 0.05). BK: basal diet, SS: sodium selenite, SY: selenium yeast, SYC: selenium-enriched yeast culture, Se L: selenium levels, and Se S: selenium sources. ^1^ HS− = Normal temperature (26 ± 2 °C), HS+ = Circulating high temperature (26 ± 2 °C~33 ± 2 °C). ^2^ Weeks 45–48, 49–50, and 51–52: each group has six replicates (*n* = 6).

**Table 5:** Effects of different Se sources in diet on Se and VE content in serum of laying hens.

Diets	HS ^1^	Se (µg/mL)			VE (µg/mL)		
		Week 48 ^2^	Week 50 ^2^	Week 52 ^2^	Week 48	Week 50	Week 52
BK	−	0.08	0.07	0.06 ^d^	75.94	81.51 ^c^	81.71 ^b,c^
BK	+	0.07	0.04	0.06 ^d^	113.38	34.91 ^a^	70.60 ^c^
SS	−	0.18	0.17	0.18 ^a,b^	61.61	84.97 ^b,c^	106.15 ^a,b^
SS	+	0.19	0.13	0.15 ^c^	75.92	42.99 ^a^	94.54 ^a,b,c^
SY	−	0.19	0.18	0.20 ^a^	90.81	83.39 ^a,b^	79.20 ^b,c^
SY	+	0.18	0.15	0.16 ^b,c^	75.46	66.38 ^a^	102.32 ^a,b^
SYC	−	0.19	0.18	0.19 ^a,b^	123.97	77.77 ^a^	87.85 ^a,b,c^
SYC	+	0.18	0.15	0.16 ^b,c^	61.37	75.40 ^a^	116.36 ^a^
SEM		0.004	0.007	0.005	10.800	5.016	8.114
BK		0.08 ^b^	0.06 ^b^	0.06 ^b^	94.66	58.21	76.16 ^b^
SS		0.18 ^a^	0.15 ^a^	0.17 ^a^	68.77	63.98	100.34 ^a,b^
SY		0.19 ^a^	0.17 ^a^	0.18 ^a^	83.14	74.89	90.76 ^a,b^
SYC		0.18 ^a^	0.16 ^a^	0.18 ^a^	92.67	76.59	102.10 ^a^
SEM		0.008	0.005	0.003	7.636	3.547	5.737
	–	0.16	0.15 ^a^	0.16 ^a^	88.08	81.91 ^a^	88.73
	+	0.16	0.12 ^b^	0.13 ^b^	81.53	54.92 ^b^	95.95
SEM		0.002	0.003	0.002	5.400	2.508	4.057
*p*-values	HS	0.137	0.008	<0.001	0.397	<0.001	0.350
	Se L	<0.001	<0.001	<0.001	0.146	0.073	0.002
	Se S	0.421	0.125	0.018	0.099	0.241	0.335
	Se L × HS	0.595	0.991	<0.001	0.002	0.003	0.074
	Se S × HS	0.264	0.642	0.549	0.004	0.002	0.039

Different letters indicate statistically significant differences (*p* < 0.05). BK: basal diet, SS: sodium selenite, SY: selenium yeast, SYC: selenium-enriched yeast culture, VE: Vitamin E, Se L: selenium levels, and Se S: selenium sources. ^1^ HS− = Normal temperature (26 ± 2 °C), HS+ = Circulating high temperature (26 ± 2 °C~33 ± 2 °C). ^2^ Weeks 48, 50, and 52: each group has six replicates (*n* = 6).

**Table 6:** Effects of dietary different Se sources on serum antioxidant status of laying hens.

Diets	HS ^1^	MDA (nmol/mL)			GSH-Px (U/mL)			Nrf-2(ng/mL)			Keap-1(pg/mL)		
		Week 48 ^2^	Week 50 ^2^	Week 52 ^2^	Week 48	Week 50	Week 52	Week 48	Week 50	Week 52	Week 48	Week 50	Week 52
BK	−	3.99	5.42	4.30	368.37	283.82	292.40	3.32	2.46	2.67	141.44	105.42	93.27
BK	+	3.89	5.32	4.14	406.02	294.61	319.71	3.30	2.36	2.60	169.22	117.00	113.48
SS	−	2.62	3.91	2.78	445.43	387.30	449.84	3.27	2.25	2.33	113.67	94.82	97.95
SS	+	2.00	4.06	3.26	452.70	417.47	492.77	3.24	2.19	1.90	142.77	107.21	89.39
SY	−	2.72	4.08	3.28	442.80	406.90	418.35	3.77	2.29	2.08	168.31	89.75	99.52
SY	+	2.40	4.20	3.60	443.23	389.73	431.34	3.47	1.69	2.41	128.98	76.82	102.09
SYC	−	3.03	4.31	4.09	438.39	375.12	219.30	3.44	1.92	2.40	122.21	91.24	96.33
SYC	+	3.50	4.39	4.36	437.87	365.91	335.56	2.79	2.63	2.38	146.08	114.43	100.39
SEM		0.317	0.270	0.296	11.027	23.078	26.699	0.193	0.273	0.313	13.703	9.594	6.326
BK		3.94 ^a^	5.37 ^a^	4.22 ^a^	387.19 ^b^	289.21 ^b^	306.06 ^b^	3.31	2.41	2.63	155.33	111.21 ^a^	103.37
SS		2.31 ^c^	3.98 ^b^	3.02 ^b^	449.06 ^a^	402.39 ^a^	471.30 ^a^	3.25	2.22	2.12	128.22	101.02 ^ab^	93.67
SY		2.56 ^c^	4.14 ^b^	3.44 ^b^	443.01 ^a^	398.31 ^a^	424.84 ^a^	3.62	1.99	2.25	148.65	83.28 ^b^	100.81
SYC		3.26 ^ab^	4.35 ^b^	4.22 ^a^	438.13 ^a^	370.51 ^a^	277.43 ^b^	3.11	2.28	2.39	134.15	102.84 ^ab^	98.36
SEM		0.148	0.191	0.209	5.514	16.319	18.879	0.074	0.193	0.221	5.317	6.784	4.473
	–	3.09	4.43	3.61	423.74	363.28	344.97 ^b^	3.45	2.23	2.37	136.41	95.31	96.77
	+	2.94	4.49	3.84	434.95	366.93	394.84 ^a^	3.20	2.22	2.32	146.76	103.87	101.34
SEM		0.158	0.135	0.148	5.513	11.539	13.349	0.096	0.137	0.156	6.852	4.797	3.163
*p*-values	HS	0.520	0.752	0.159	0.160	0.845	0.020	0.077	0.965	0.887	0.293	0.281	0.385
	Se L	<0.001	<0.001	0.010	<0.001	<0.001	<0.001	0.895	0.278	0.146	0.111	0.057	0.273
	Se S	0.015	0.406	0.001	0.615	0.336	<0.001	0.054	0.549	0.676	0.322	0.095	0.525
	Se L × HS	0.921	0.636	0.297	0.059	0.802	0.495	0.339	0.797	0.949	0.307	0.799	0.052
	Se S × HS	0.222	0.990	0.929	0.929	0.553	0.155	0.279	0.068	0.483	0.695	0.171	0.558

Different letters indicate statistically significant differences (*p* < 0.05). BK: basal diet, SS: sodium selenite, SY: selenium yeast, SYC: selenium-enriched yeast culture, MDA: malondialdehyde:, GSH-Px: glutathione peroxidase, Nrf-2: nuclear factor-E2-related factor-2, Keap-1: Kelch-like ECH-associated protein-1, Se L: selenium levels, and Se S: selenium sources. ^1^ HS− = Normal temperature (26 ± 2 °C), HS+ = Circulating high temperature (26 ± 2 °C~33 ± 2 °C). ^2^ Weeks 48, 50, and 52: each group has six replicates (*n* = 6).

**Table 7:** Effects of different Se sources in diet on serum immune indexes of laying hens.

Diets	HS ^1^	IgA (g/L)			IgG (g/L)			IgM (g/L)			IL-1β (pg/mL)			IL-6 (pg/mL)			IL-10 (pg/mL)		
		Week 48 ^2^	Week 50 ^2^	Week 52 ^2^	Week 48	Week 50	Week 52	Week 48	Week 50	Week 52	Week 48	Week 50	Week 52	Week 48	Week 50	Week 52	Week 48	Week 50	Week 52
BK	−	1.06	0.92	1.11	6.71	6.12	6.42	0.73	0.55	0.74	26.85	31.99	32.48	86.00	86.69	85.90	32.82	19.95	29.97
BK	+	1.04	0.86	1.09	7.58	5.23	5.19	0.68	0.56	0.78	28.88	33.39	27.43	89.56	86.35	89.65	32.85	22.16	28.58
SS	−	0.96	0.91	1.08	6.28	6.51	8.91	0.58	0.60	0.73	27.92	31.08	28.19	85.19	86.97	75.14	31.28	20.41	26.80
SS	+	1.05	0.84	1.31	6.92	6.12	7.04	0.71	0.56	0.94	25.15	26.20	24.80	82.30	84.89	78.74	28.27	22.31	26.45
SY	−	0.99	1.03	1.03	7.69	7.33	6.53	0.66	0.68	0.79	22.18	28.87	30.66	91.11	75.18	77.74	32.90	21.04	28.16
SY	+	1.25	0.99	1.08	7.08	5.94	5.97	0.69	0.59	0.66	28.61	28.33	29.40	86.03	83.85	74.44	27.66	23.83	26.09
SYC	−	1.03	1.06	1.03	6.71	6.05	6.77	0.76	0.55	0.88	25.21	27.49	28.14	82.74	87.08	79.76	29.30	25.36	27.93
SYC	+	1.11	0.83	1.04	7.23	4.76	6.87	0.80	0.45	0.67	26.30	30.36	25.61	79.53	88.28	75.67	27.22	21.33	23.35
SEM		0.094	0.067	0.098	0.568	0.544	0.674	0.049	0.050	0.065	1.711	1.979	1.599	2.855	3.889	3.835	1.726	1.159	1.369
BK		1.05	0.89	1.10	7.15	5.67	5.81 ^b^	0.71 ^a,b^	0.55 ^a,b^	0.76	27.87	32.69	29.95	87.78 ^a^	86.52	87.78 ^a^	32.83	21.05	29.27
SS		1.00	0.87	1.19	6.60	6.31	7.97 ^a^	0.65 ^b^	0.58 ^ab^	0.83	26.53	28.64	26.50	83.74 ^a,b^	85.93	76.94 ^b^	29.77	21.36	26.63
SY		1.12	1.01	1.06	7.38	6.63	6.25 ^a,b^	0.67 ^a,b^	0.63 ^a^	0.72	25.40	28.60	30.03	88.57 ^a^	79.52	76.09 ^b^	30.28	22.43	27.12
SYC		1.07	0.94	1.03	6.97	5.41	6.82 ^a,b^	0.78 ^a^	0.50 ^b^	0.77	25.75	28.93	26.87	81.14 ^b^	87.68	77.72 ^b^	28.26	23.35	25.64
SEM		0.033	0.047	0.069	0.195	0.385	0.477	0.018	0.035	0.046	0.641	1.399	1.131	1.078	2.750	2.712	0.664	0.819	0.968
	−	1.01	0.98	1.06	6.85	6.51 ^a^	7.16	0.68	0.59	0.78	25.54	29.86	29.87 ^a^	86.26	83.98	79.64	31.58	21.69	28.22 ^a^
	+	1.11	0.88	1.13	7.20	5.51 ^b^	6.27	0.72	0.54	0.76	27.24	29.57	26.81 ^b^	84.36	85.84	79.63	29.00	22.41	26.11 ^b^
SEM		0.047	0.033	0.049	0.284	0.272	0.337	0.025	0.025	0.033	0.856	0.990	0.800	1.427	1.944	1.918	0.863	0.579	0.684
*p*-values	HS	0.140	0.226	0.239	0.385	0.012	0.196	0.305	0.312	0.525	0.171	0.860	0.013	0.353	0.284	0.997	0.043	0.387	0.037
	Se L	0.826	0.338	0.911	0.727	0.325	0.036	0.865	0.675	0.769	0.168	0.020	0.109	0.167	0.503	0.001	0.022	0.171	0.017
	Se S	0.490	0.146	0.222	0.393	0.080	0.046	0.035	0.041	0.280	0.795	0.984	0.067	0.042	0.103	0.914	0.482	0.245	0.551
	Se L × HS	0.301	0.579	0.486	0.469	0.880	0.683	0.176	0.289	0.468	0.875	0.491	0.317	0.128	0.647	0.429	0.226	0.299	0.676
	Se S × HS	0.576	0.303	0.499	0.482	0.604	0.342	0.584	0.780	0.513	0.257	0.162	0.800	0.918	0.378	0.551	0.647	0.842	0.312

Different letters indicate statistically significant differences (*p* < 0.05). BK: basal diet, SS: sodium selenite, SY: selenium yeast, SYC: selenium-enriched yeast culture, IgA: immunoglobulin A, IgG: immunoglobulin G, IgM: immunoglobulin M, IL-1β: interleukin-1β, IL-6: interleukin-6, IL-10: interleukin-10, Se L: selenium levels, and Se S: selenium sources. ^1^ HS− = Normal temperature (26 ± 2 °C), HS+ = Circulating high temperature (26 ± 2 °C~33 ± 2 °C). ^2^ Weeks 48, 50, and 52: each group has six replicates (*n* = 6).

**Table 8:** Effects of different Se sources in diets on serum heat stress indexes of laying hens.

Diets	HS ^1^	FT3 (pmol/L)			FT4 (pmol/L)			FT4 (pmol/L)			CORT (nmol/L)			HSPs (ng/L)		
		Week 48 ^2^	Week 50 ^2^	Week 52 ^2^	Week 48	Week 50	Week 52	Week 48	Week 50	Week 52	Week 48	Week 50	Week 52	Week 48	Week 50	Week 52
BK	−	1.88	1.98	2.01	3.77	4.08	4.78	9.23	8.89	8.80 ^b^	130.29	112.52	103.97	329.02	418.15	274.16
BK	+	2.09	2.72	1.78	4.05	4.61	3.7	10.56	9.65	14.03 ^a^	118.44	115.89	121.52	286.24	404.79	302.08
SS	−	2.06	2.26	2.31	3.54	5.06	4.65	11.91	10.34	8.41 ^b^	124.32	121.89	118.74	324.65	359.57	253.50
SS	+	1.72	2.76	1.88	3.66	5.99	3.35	9.69	8.14	12.30 ^a^	117.12	108.13	108.58	258.34	370.66	267.52
SY	−	1.81	2.62	2.64	3.68	6.15	4.74	10.08	8.47	13.79 ^a^	112.01	105.75	100.91	274.20	365.90	222.89
SY	+	2.16	3.31	2.95	3.69	6.33	4.63	9.2	8.11	13.53 ^a^	125.63	123.89	112.38	273.45	407.63	273.09
SYC	−	1.75	2.30	2.14	4.06	5.08	4.08	9.12	8.90	12.86 ^a^	112.76	116.08	103.84	251.98	352.42	245.70
SYC	+	1.63	3.15	1.96	4.18	5.60	6.27	9.10	8.90	13.06 ^a^	113.24	118.52	107.81	233.90	392.82	314.50
SEM		0.194	0.311	0.276	0.515	0.803	0.645	0.735	0.590	0.553	5.326	4.970	4.737	19.836	28.604	17.023
BK		1.99	2.35	1.89 ^b^	3.91	4.35	4.24	9.90	9.27	11.41 ^a,b^	124.37	114.20	112.74	307.63	411.47	288.12
SS		1.89	2.51	2.10 ^a,b^	3.60	5.53	4.00	10.80	9.24	10.35 ^b^	120.72	115.01	113.66	291.49	365.11	260.51
SY		1.98	2.97	2.79 ^a^	3.69	6.24	4.69	9.64	8.29	13.66 ^a^	118.82	114.82	106.64	273.82	386.77	247.99
SYC		1.69	2.72	2.05 ^b^	4.12	5.34	5.18	9.11	8.90	12.96 ^a^	113.00	117.30	105.83	242.94	372.62	280.10
SEM		0.137	0.220	0.195	0.364	0.568	0.456	0.520	0.417	0.391	3.766	3.514	3.349	14.026	20.226	12.037
	–	1.88	2.29 ^b^	2.28	3.76	5.09	4.56	10.08	9.15	10.96 ^b^	119.84	114.06	106.86	294.96 ^a^	374.01	249.07 ^b^
	+	1.90	2.99 ^a^	2.14	3.90	5.64	4.49	9.64	8.70	13.23 ^a^	118.61	116.60	112.57	262.98 ^b^	393.98	289.30 ^a^
SEM		0.097	0.156	0.138	0.257	0.401	0.323	0.368	0.295	0.277	2.663	2.485	2.368	9.918	14.302	8.511
*p*-values	HS	0.848	0.005	0.488	0.707	0.377	0.867	0.396	0.357	0.004	0.745	0.474	0.128	0.029	0.4	0.015
	Se L	0.858	0.143	0.071	0.819	0.046	0.476	0.161	0.347	0.052	0.101	0.713	0.305	0.292	0.127	0.079
	Se S	0.905	0.361	0.018	0.219	0.502	0.203	0.268	0.279	<0.001	0.808	0.858	0.208	0.285	0.746	0.18
	Se L × HS	0.928	0.905	0.787	0.649	0.991	0.211	0.09	0.104	<0.001	0.091	0.894	0.05	0.528	0.349	0.559
	Se S × HS	0.529	0.854	0.402	0.915	0.895	0.033	0.273	0.151	0.001	0.418	0.012	0.083	0.798	0.833	0.276

Different letters indicate statistically significant differences (*p* < 0.05). BK: basal diet, SS: sodium selenite, SY: selenium yeast, SYC: selenium-enriched yeast culture, FT3: free triiodothyronine, FT4: free thyroxine, DI-I: deiodinase-I, CORT: corticosterone, HSPs: heat stress protein, Se L: selenium levels, and Se S: selenium sources. ^1^ HS− = Normal temperature (26 ± 2 °C), HS+ = Circulating high temperature (26 ± 2 °C~33 ± 2 °C). ^2^ Weeks 48, 50, and 52: each group has six replicates (*n* = 6).

## Data Availability

The data presented in this study are available on request from the corresponding author.

## Supplementary Materials

The following supporting information can be downloaded at: https://www.mdpi.com/article/10.3390/ani12081006/s1. Figure S1: Changes in rectal temperature of laying hens of different periods. BK: basal diet, SS: sodium selenite, SY: selenium yeast, SYC: selenium-enriched yeast culture. HS+ = under circulating high temperature (26 ± 2 °C~33 ± 2 °C). Significance compared with 48 week, * *p* < 0.05, ** *p* < 0.01 and *** *p* < 0.001, # indicated that 52 week was compared with 50 week, # *p* < 0.05, ## *p* < 0.01, ### *p* < 0.001. Figure S2: Changes in laying performance of laying hens of different periods. BK: basal diet, SS: sodium selenite, SY: selenium yeast, SYC: selenium-enriched yeast culture. HS+ = under circulating high temperature (26 ± 2 °C~33 ± 2 °C). Significance compared with 45–48 weeks, * *p* < 0.05, ** *p* < 0.01 and *** *p* < 0.001, # indicated that 51–52 weeks was compared with 49–50 weeks, # *p* < 0.05, ## *p* < 0.01, ### *p* < 0.001. Figure S3. Changes in egg quality of laying hens of different periods. BK: basal diet, SS: sodium selenite, SY: selenium yeast, SYC: selenium-enriched yeast culture. HS+ = under circulating high temperature (26 ± 2 °C~33 ± 2 °C). Significance compared with 45–48 weeks, * *p* < 0.05, ** *p* < 0.01 and *** *p* < 0.001, # indicated that 51–52 weeks was compared with 49–50 weeks, # *p* < 0.05, ## *p* < 0.01, ### *p* < 0.001. Figure S4. Changes in Se and VE content in serum of laying hens of different periods. BK: basal diet, SS: sodium selenite, SY: selenium yeast, SYC: selenium-enriched yeast culture. HS+ = under circulating high temperature (26 ± 2 °C~33 ± 2 °C). Significance compared with 48 week, * *p* < 0.05, ** *p* < 0.01 and *** *p* < 0.001, # indicated that 52 week was compared with 50 week, # *p* < 0.05, ## *p* < 0.01, ### *p* < 0.001. Figure S5. Changes in antioxidant status of laying hens of different periods. BK: basal diet, SS: sodium selenite, SY: selenium yeast, SYC: selenium-enriched yeast culture. HS+ = under circulating high temperature (26 ± 2 °C~33 ± 2 °C). Significance compared with 48 week, * *p* < 0.05, ** *p* < 0.01 and *** *p* < 0.001, # indicated that 52 week was compared with 50 week, # *p* < 0.05, ## *p* < 0.01, ### *p* < 0.001. Figure S6. Changes in serum immune indexes of laying hens of different periods. BK: basal diet, SS: sodium selenite, SY: selenium yeast, SYC: selenium-enriched yeast culture. HS+ = under circulating high temperature (26 ± 2 °C~33 ± 2 °C). Significance compared with 48 week, * *p* < 0.05, ** *p* < 0.01 and *** *p* < 0.001, # indicated that 52 week was compared with 50 week, # *p* < 0.05, ## *p* < 0.01, ### *p* < 0.001. Figure S7. Changes in serum heat stress indexes of laying hens of different periods. BK: basal diet, SS: sodium selenite, SY: selenium yeast, SYC: selenium-enriched yeast culture. HS+ = under circulating high temperature (26 ± 2 °C~33 ± 2 °C). Significance compared with 48 week, * *p* < 0.05, ** *p* < 0.01 and *** *p* < 0.001, # indicated that 52 week was compared with 50 week, # *p* < 0.05, ## *p* < 0.01, ### *p* < 0.001.

## References

[1] Mashaly M.M., Hendricks G.L., Kalama M.A., Gehad A.E., Patterson P.H. (2004). Effect of Heat Stress on Production Parameters and Immune Responses of Commercial Laying Hens. Poult. Sci..

[2] Battisti D.S., Naylor R.L. (2009). Historical warnings of future food insecurity with unprecedented seasonal heat. Science.

[3] Mack L.A., Felver-Gant J.N., Dennis R.L., Cheng H.W. (2013). Genetic variations alter production and behavioral responses following heat stress in 2 strains of laying hens. Poult. Sci..

[4] Li W.X., Chen Y.Q., Zhao L.H., Ma Q.G., Zhang J.Y., Ji C. (2018). No copper supplementation in a corn-soybean basal diet has no adverse effects on late-phase laying hens under normal and cyclic high temperatures. Poult. Sci..

[5] Sun X., Yue S., Qiao Y. (2020). Dietary supplementation with selenium-enriched earthworm powder improves antioxidative ability and immunity of laying hens. Poult. Sci..

[6] Rayman M.P. (2000). The importance of selenium to human health. Lancet.

[7] Noguchi T., Cantor A.H., Scott M.L. (1973). Mode of action of selenium and vitamin E in prevention of exudative diathesis in chicks. J. Nutr..

[8] Whitacre M.E., Combs G.F. (1983). Selenium and mitochondrial integrity in the pancreas of the chick. J. Nutr..

[9] Whanger P.D., Weswig P.H., Schmitz J.A., Oldfield J.E. (1977). Effects of selenium and vitamin E on blood selenium levels, tissue glutathione peroxidase activities and white muscle disease in sheep fed purified or hay diets. J. Nutr..

[10] Suhajda A., Hegoczki J., Janzso B., Pais I., Vereczkey G. (2000). Preparation of selenium yeasts I. Preparation of selenium-enriched Saccharomyces cerevisiae. J. Trace Elem. Med. Biol..

[11] Payne R.L., Lavergne T.K., Southern L.L. (2005). Effect of inorganic versus organic selenium on hen production and egg selenium concentration. Poult. Sci..

[12] Yoon I., Werner T.M., Butler J.M. (2007). Effect of source and concentration of selenium on growth performance and selenium retention in broiler chickens. Poult. Sci..

[13] Lu J., Qu L., Ma M., Li Y.F., Wang X.G., Yang Z., Wang K.H. (2020). Efficacy evaluation of selenium-enriched yeast in laying hens: Effects on performance, egg quality, organ development, and selenium deposition. Poult. Sci..

[14] Lu J., Qu L., Shen M.M., Wang X.G., Guo J., Hu Y.P., Dou T.C., Wang K.H. (2019). Effects of high-dose selenium-enriched yeast on laying performance, egg quality, clinical blood parameters, organ development, and selenium deposition in laying hens. Poult. Sci..

[15] Mahmoud K.Z., Edens F.W. (2005). Influence of organic selenium on hsp70 response of heat-stressed and enteropathogenic Escherichia coli-challenged broiler chickens (*Gallus gallus*). Comp. Biochem. Physiol..

[16] Kim Y.Y., Mahan D.C. (2001). Comparative effects of high dietary levels of organic and inorganic selenium on selenium toxicity of growing-finishing pigs. J. Anim. Sci..

[17] Hoffman D.J. (2002). Role of selenium toxicity and oxidative stress in aquatic birds. Aquat. Toxicol..

[18] Pan C., Zhao Y., Liao S.F. (2011). Effect of selenium-enriched probiotics on laying performance, egg quality, egg selenium content, and egg glutathione peroxidase activity. J. Agric. Food Chem..

[19] Liao X.D., Lu L., Li S.F., Liu S.B., Zhang L.Y., Wang G.Y., Lu A., Luo X.G. (2012). Effects of selenium source and level on growth performance, tissue selenium concentrations, antioxidation, and immune functions of heat-stressed broilers. Biol. Trace Element Res..

[20] Barrett N.W., Rowland K., Schmidt C.J., Lamont S.J., Rothschild M.F., Ashwell C.M. (2019). Effects of acute and chronic heat stress on the performance, egg quality, body temperature, and blood gas parameters of laying hens. Poult. Sci..

[21] Star L., Juul-Madsen H.R., Decuypere E., Nieuwland M., de Vries Reilingh G., Van Den Brand H., Kemp B., Parmentier H.K. (2009). Effect of early life thermal conditioning and immune challenge on thermotolerance and humoral immune competence in adult laying hens. Poult. Sci..

[22] Januzi V., Sena L., Elezi X. (2017). The influence of layers feed supplementation with organic selenium on the eggs quality and selenium content in the egg. Albanian J. Agric. Sci..

[23] Ebeid T.A., Suzuki T., Sugiyama T. (2012). High ambient temperature influences eggshell quality and calbindin-D28k localization of eggshell gland and all intestinal segments of laying hens. Poult. Sci..

[24] Song Z., Liu L., Sheikhahmadi A. (2012). Effect of heat exposure on gene expression of feed intake regulatory peptides in laying hens. J. Biomed. Biotechnol..

[25] Morales A., Hernández L., Buenabad L. (2016). Effect of heat stress on the endogenous intestinal loss of amino acids in growing pigs. J. Anim. Sci..

[26] Tadtiyanant C., Lyons J.J., Vandepopuliere J.M. (1991). Influence of wet and dry feed on laying hens under heat stress. Poult. Sci..

[27] Tanor M.A., Leeson S., Summers J.D. (1984). Effect of heat stress and diet composition on performance of White Leghorn hens. Poult. Sci..

[28] Paton N.D. (2001). Organic Selenium in the Nutrition of Laying Hens: Effects on Egg Selenium Content, Egg Quality and Transfer to Developing Chick Embryo. Ph.D. Thesis.

[29] Lin H., Decuypere E., Buyse J. (2006). Acute heat stress induces oxidative stress in broiler chickens. Comp. Biochem. Physiol. A..

[30] Belhadj Slimen I., Najar T., Ghram A., Dabbebi H., Ben Mrad M., Abdrabbah M. (2014). Reactive oxygen species, heat stress and oxidative-induced mitochondrial damage. A review. Int. J. Hyperth..

[31] Jones D.P. (2002). Redox potential of GSH/GSSG couple: Assay and biological significance. Method Enzymol..

[32] Dalia A.M., Loh T.C., Sazili A.Q., Jahromi M.F., Samsudin A.A. (2017). The effect of dietary bacterial organic selenium on growth performance, antioxidant capacity, and Selenoproteins gene expression in broiler chickens. BMC Veter Res..

[33] Han X.J., Qin P., Li W.X., Ma Q.G., Ji C., Zhang J.Y., Zhao L.H. (2017). Effect of sodium selenite and selenium yeast on performance, egg quality, antioxidant capacity, and selenium deposition of laying hens. Poult. Sci..

[34] Rotruck J.T., Pope A.L., Ganther H.E. (1973). Selenium: Biochemical role as a component of glutathione peroxidase. Science.

[35] Tirawattanawanich C., Chantakru S., Nimitsantiwong W., Tongyai S. (2011). The effects of tropical environmental conditions on the stress and immune responses of commercial broilers, Thai indigenous chickens, and crossbred chickens. J. Appl. Poult. Res..

[36] Lara L.J., Rostagno M.H. (2013). Impact of heat stress on poultry production. Animals.

[37] Park S.O., Hwangbo J.H., Ryu C.M., Park B.S., Choi Y.H. (2013). Effects of Extreme Heat Stress on Growth Performance, Lymphoid Organ, IgG and Cecum Microflora of Broiler Chickens. Int. J. Agric. Biol..

[38] Vakili R., Bahram M. (2010). Effects of different dietary levels of selenium on metabolic parameters and humoral immunity in broiler chickens. J. Vet. Res..

[39] Antoni F.A. (1986). Hypothalamic control of adrenocorticotropin secretion: Advances since the discovery of 41-residue corticotropin-releasing factor. Endocr. Rev..

[40] Dallman M.F., Akana S.F., Jacobson L., Levin N., Cascio C.S., Shinsako J. (1987). Characterization of Corticosterone Feedback Regulation of ACTH Secretion. Ann. N. Y. Acad. Sci..

[41] Vale W., Spiess J., Rivier C., Rivier J. (1981). Characterization of a 41-residue ovine hypothalamic peptide that stimulates secretion of corticotropin and β-endorphin. Science.

[42] Quinteiro-Filho W.M., Ribeiro A., Ferraz-de-Paula V., Pinheiro M.L., Sakai M., Sá L., Ferreira A., Palermo-Neto J. (2010). Heat stress impairs performance parameters, induces intestinal injury, and decreases macrophage activity in broiler chickens. Poult. Sci..

[43] He X., Lu Z., Ma B., Zhang L., Li J. (2019). Chronic heat stress alters hypothalamus integrity, the serum indexes and attenuates expressions of hypothalamic appetite genes in broilers. J. Therm. Biol..

