# Supplementary material for: Effects of Different Selenium Sources on the Laying Performance, Egg Quality, Antioxidant, and Immune Responses of Laying Hens under Normal and Cyclic High Temperatures
Source: Animals (Basel). 2022 Apr 13;12(8):1006. doi: 10.3390/ani12081006 (PMC9028492; doi:10.3390/ani12081006)
Supplement: Supplementary file 1 [file animals-12-01006-s001.zip › animals-1618024-supplementary.pdf]

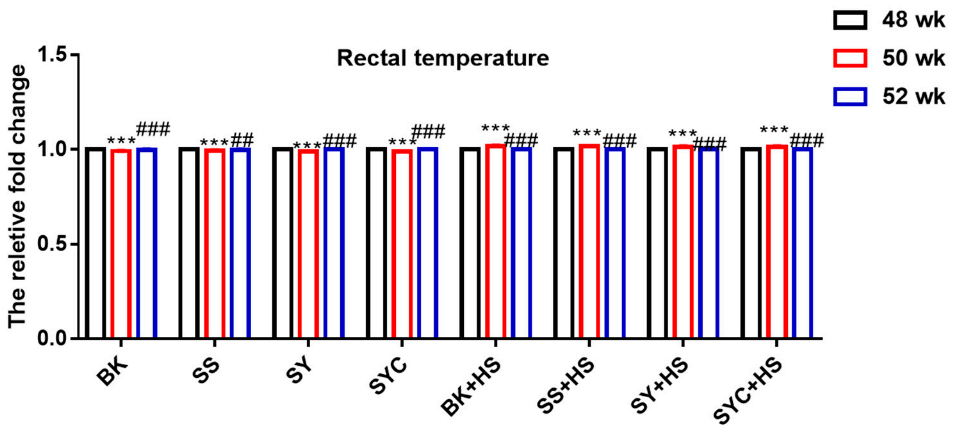

Figure S1. Changes in rectal temperature of laying hens of different periods. BK: basal diet, SS: sodium selenite, SY: selenium yeast, SYC: selenium-enriched yeast culture. +HS = under circulating high temperature (26 ± 2 ℃~33±2 ℃). Significance compared with 48 wk, * $P < 0.05$, ** $P < 0.01$ and *** $P < 0.001$, # indicated that 52 wk was compared with 50 wk, # $P < 0.05$., ## $P < 0.01$, ###$P < 0.001$.

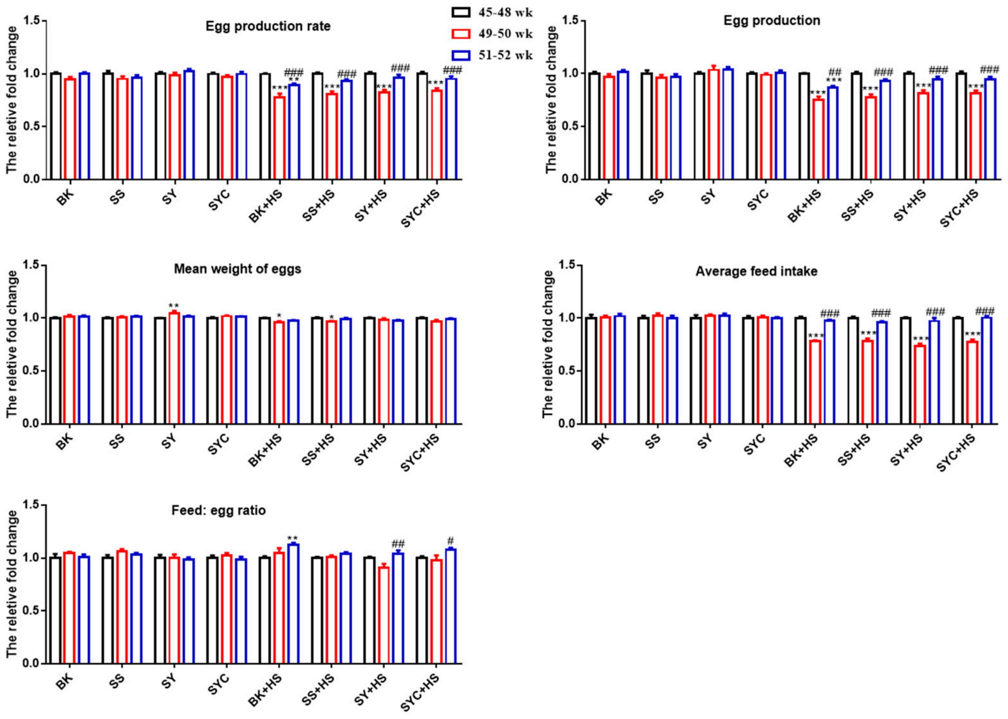

Figure S2. Changes in laying performance of laying hens of different periods. BK: basal diet, SS: sodium selenite, SY: selenium yeast, SYC: selenium-enriched yeast culture. +HS = under circulating high temperature (26 ± 2 ℃~33±2 ℃). Significance compared with 45-48 wk, * $P < 0.05$, ** $P < 0.01$ and *** $P < 0.001$, # indicated that 51-52 wk was compared with 49-50 wk, # $P < 0.05$., ## $P < 0.01$, ###$P < 0.001$

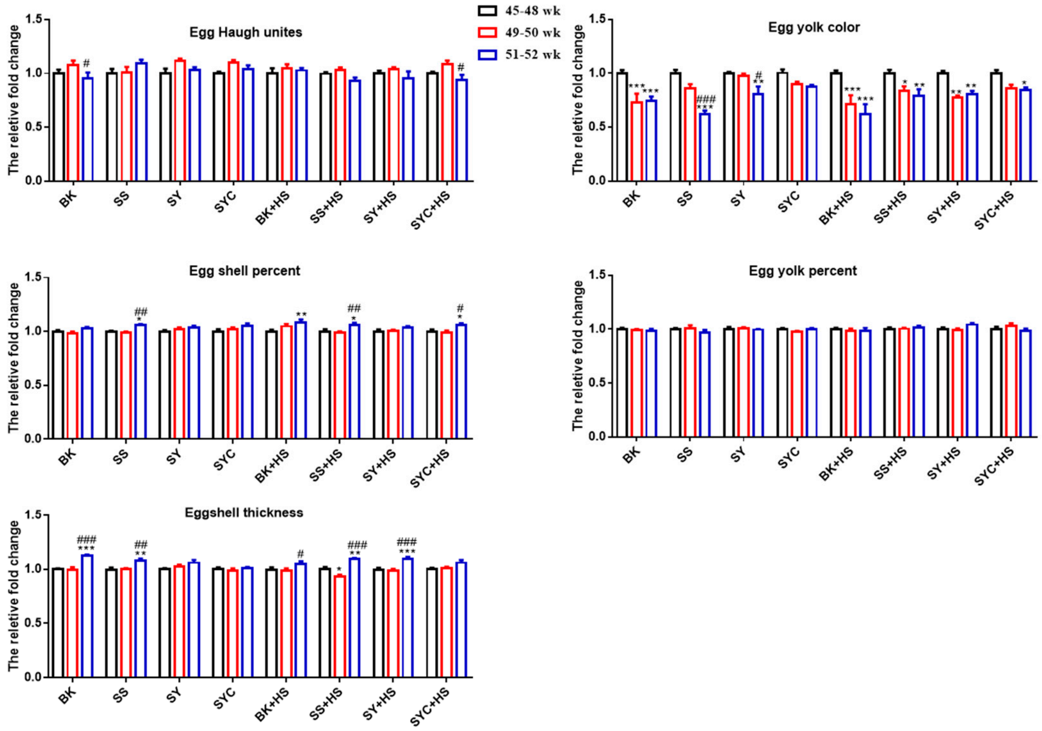

Figure S3. Changes in egg quality of laying hens of different periods. BK: basal diet, SS: sodium selenite, SY: selenium yeast, SYC: selenium-enriched yeast culture. +HS = under circulating high temperature (26 ± 2 ℃~33±2 ℃). Significance compared with 45-48 wk, * $P < 0.05$, ** $P < 0.01$ and *** $P < 0.001$, # indicated that 51-52 wk was compared with 49-50 wk, # $P < 0.05$., ## $P < 0.01$, ###$P < 0.001$.

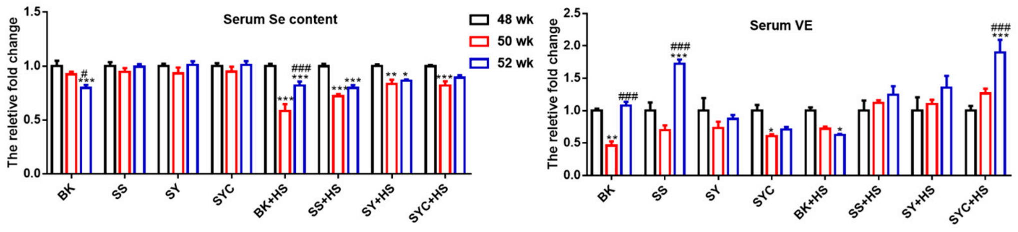

Figure S4. Changes in Se and VE content in serum of laying hens of different periods. BK: basal diet, SS: sodium selenite, SY: selenium yeast, SYC: selenium-enriched yeast culture. +HS = under circulating high temperature (26 ± 2 ℃~33±2 ℃). Significance compared with 48 wk, * $P < 0.05$, ** $P < 0.01$ and *** $P < 0.001$, # indicated that 52 wk was compared with 50 wk, # $P < 0.05$., ## $P < 0.01$, ###$P < 0.001$.

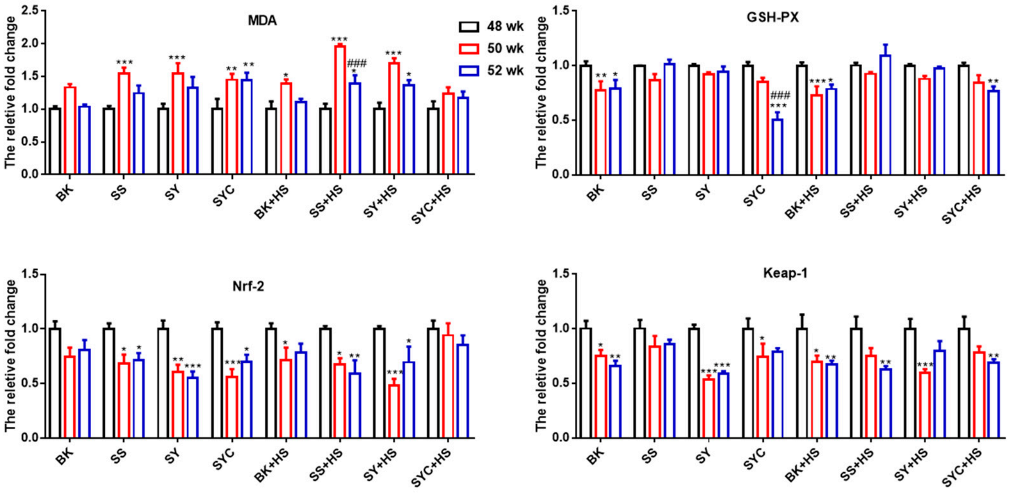

Figure S5. Changes in antioxidant status of laying hens of different periods. BK: basal diet, SS: sodium selenite, SY: selenium yeast, SYC: selenium-enriched yeast culture. +HS = under circulating high temperature (26 ± 2 ℃~33±2 ℃). Significance compared with 48 wk, * P < 0.05, ** P < 0.01 and *** P < 0.001, # indicated that 52 wk was compared with 50 wk, # P < 0.05., ## P < 0.01, ###P < 0.001.

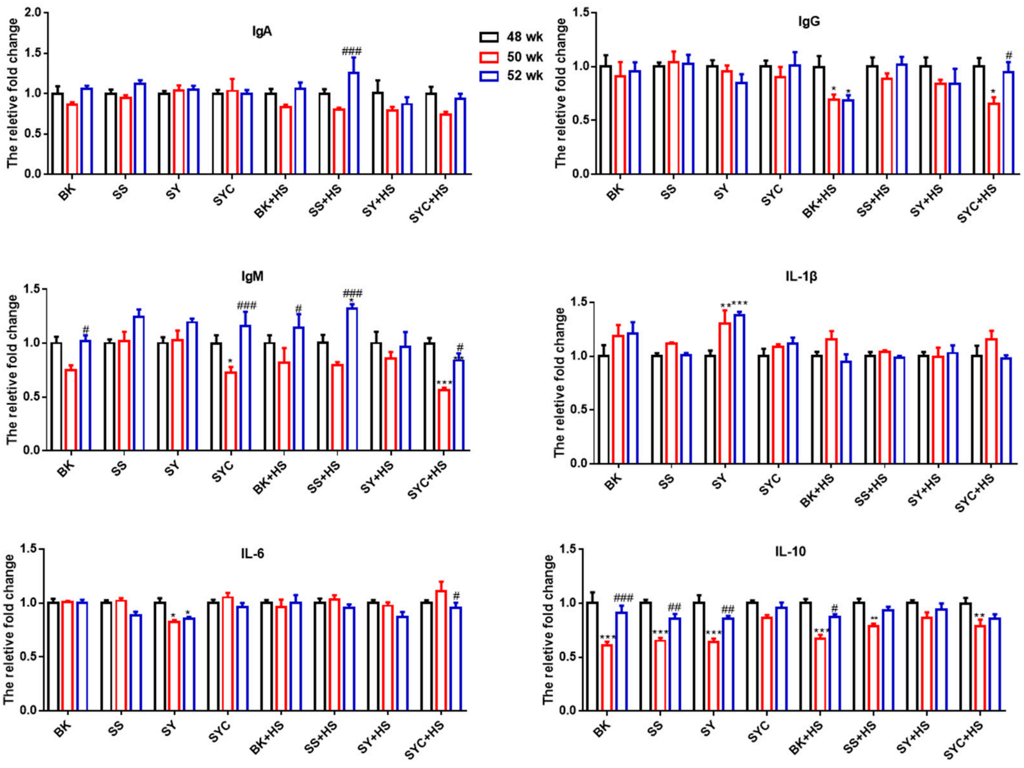

Figure S6. Changes in serum immune indexes of laying hens of different periods. BK: basal diet, SS: sodium selenite, SY: selenium yeast, SYC: selenium-enriched yeast culture. +HS = under circulating high temperature (26 ± 2 ℃~33±2 ℃). Significance compared with 48 wk, * $P < 0.05$, ** $P < 0.01$ and *** $P < 0.001$, # indicated that 52 wk was compared with 50 wk, # $P < 0.05$., ## $P < 0.01$, ###$P < 0.001$.

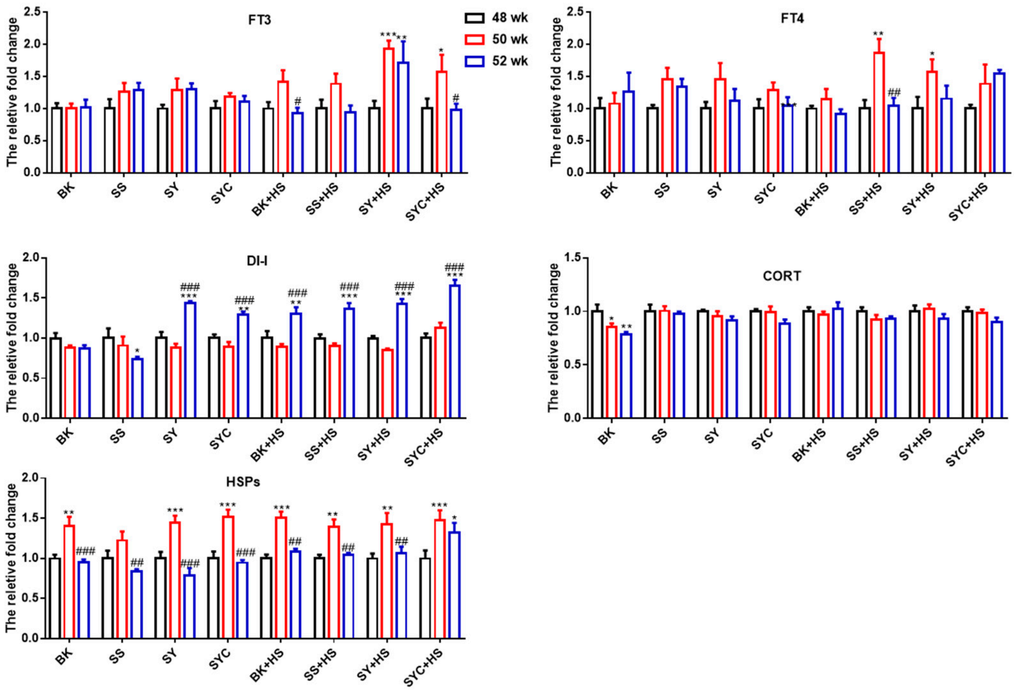

Figure S7. Changes in serum heat stress indexes of laying hens of different periods. BK: basal diet, SS: sodium selenite, SY: selenium yeast, SYC: selenium-enriched yeast culture. +HS = under circulating high temperature (26 ± 2 ℃~33 ± 2 ℃). Significance compared with 48 wk, * $P < 0.05$, ** $P < 0.01$ and *** $P < 0.001$, # indicated that 52 wk was compared with 50 wk, # $P < 0.05$., ## $P < 0.01$, ###$P < 0.001$.
